# Magnetic Resonance Spectroscopy-based Metabolomic Biomarkers for Typing, Staging, and Survival Estimation of Early-Stage Human Lung Cancer

**DOI:** 10.1038/s41598-019-46643-5

**Published:** 2019-07-16

**Authors:** Yannick Berker, Lindsey A. Vandergrift, Isabel Wagner, Li Su, Johannes Kurth, Andreas Schuler, Sarah S. Dinges, Piet Habbel, Johannes Nowak, Eugene Mark, Martin J. Aryee, David C. Christiani, Leo L. Cheng

**Affiliations:** 1 Department of Radiology, Massachusetts General Hospital, Harvard Medical School, Boston, Massachusetts 02114 USA; 2 Department of Pathology, Massachusetts General Hospital, Harvard Medical School, Boston, Massachusetts 02114 USA; 3 Division of X-Ray Imaging and Computed Tomography, German Cancer Research Center (DKFZ), 69120 Heidelberg, Germany; 4 Department of Urology, CCM, Charité – Universitätsmedizin Berlin, 10117 Berlin, Germany; 5 Department of Environmental Health, Harvard T.H. Chan School of Public Health and Department of Medicine, Massachusetts General Hospital/Harvard Medical School, Boston, Massachusetts 02115 USA; 6 Department of Haematology and Oncology, CCM, Charité – Universitätsmedizin Berlin, 10117 Berlin, Germany; 7 Department of Diagnostic and Interventional Radiology, University Hospital of Würzburg, 97080 Würzburg, Germany; 8 Department of Biostatistics, Harvard T.H. Chan School of Public Health, Boston, Massachusetts 02115 USA

**Keywords:** Metabolomics, Predictive markers

## Abstract

Low-dose CT has shown promise in detecting early stage lung cancer. However, concerns about the adverse health effects of radiation and high cost prevent its use as a population-wide screening tool. Effective and feasible screening methods to triage suspicious patients to CT are needed. We investigated human lung cancer metabolomics from 93 paired tissue-serum samples with magnetic resonance spectroscopy and identified tissue and serum metabolomic markers that can differentiate cancer types and stages. Most interestingly, we identified serum metabolomic profiles that can predict patient overall survival for all cases (p = 0.0076), and more importantly for Stage I cases alone (n = 58, p = 0.0100), a prediction which is significant for treatment strategies but currently cannot be achieved by any clinical method. Prolonged survival is associated with relative overexpression of glutamine, valine, and glycine, and relative suppression of glutamate and lipids in serum.

## Introduction

Despite extensive research over the past decade to improve lung cancer (LuCa) detection and treatment, LuCa presents persistent clinical challenges. The leading cause (>26%) of cancer death in the United States for both men and women, LuCa results in the number of deaths equivalent to the combination of the next four highest causes of cancer death: breast, prostate, colon, and pancreatic. LuCa is usually diagnosed at late stages, with >70% of patients dying from the disease; the ratios for breast and prostate cancer are about 16% and 17%, respectively^1^. This reality is largely attributable to the lack of a widespread, early screening test for LuCa. In its absence, the vast majority of patients seeking medical advice for symptoms of LuCa harbour locally advanced or metastatic disease.

At present, advanced radiological examinations, especially low-dose spiral CT (LDCT), can detect small LuCa lesions^2–5^. The many reports published by the National Lung Screening Trial (NLST) evaluating LDCT efficacy nonetheless question its cost-effectiveness as a screening tool^6^ and raise the issue of potential over-diagnosis^7^ and the impact of screening on quality of life^8^. Recently, the American Thoracic Society and American College of Chest Physicians published a joint official policy statement to guide the safe, effective development of LDCT screening programs^9^. Nevertheless, implementation of LDCT as a LuCa screening tool would entail considerable logistic and scientific concerns, ranging from high cost^10–13^ to, most importantly, possible radiation hazard for screened populations^14–17^. Thus, while LDCT enables detection of small lung nodules, its implementation as a screening tool for the general population is not feasible. Therefore, novel, low-cost, and safe LuCa tests that can prompt patients with suspicious screening results to seek further radiological evaluation are needed.

Current investigations of circulating blood biomarkers to develop LuCa screening methods are based on the fundamental physiology fact that all cardiac output passes through the lungs, with 20% of blood in the lungs at any given time. Thus, LuCa-associated molecules would likely be carried from lung lesions into the circulating blood. Alternatively, since metabolites in the circulating blood provide necessary nutrients for all biological and pathological processes throughout the entire body, consumption of specific metabolites by LuCa to sustain and enhance malignant processes may be measurable in blood. As a result, metabolites produced by or consumed by lung cancer lesions could serve as biomarkers.

Previously, studies of blood LuCa biomarkers have reported LuCa-associated microRNA^18–20^ and RNA fragments^21^ detected by quantitative real-time-PCR and mass spectrometry as promising markers. Inspired by the achievements in genomics, proteomics, and transcriptomics, cancer metabolomics, which reflect the functional read-outs of these upstream biological processes, can yield measures of global metabolite profiles associated with various metabolic pathways influenced by oncological developments.

To evaluate LuCa, particularly low grade LuCa and identify potential LuCa biomarkers, we investigated paired tissue and serum samples obtained from the same patients. These analyses were carried out with the special technique of high resolution magic angle spinning magnetic resonance spectroscopy (HRMAS MRS), which we developed for metabolomic analysis of intact biological tissue^22,23^ and complex fluids. This technique allows subsequent histopathology analyses of the same tissue samples, enabling spectroscopic data to be interpreted according to tissue pathologies. Since HRMAS MRS can also measure the complex biofluid of serum and obtain spectra of high resolution, tissue and serum metabolomic measures can be correlated in order to investigate the associations between metabolites of potential LuCa biomarkers measured from paired tissue and serum samples.

## Results

This study included tissue and serum pairs from 93 patients of the two major types of non-small cell LuCa (NSCLC): squamous cell carcinoma (SCC, n = 42, F = 15, M = 27, age = 67.7 ± 8.3) and adenocarcinoma (Adeno, n = 51, F = 26, M = 25, age = 63.7 ± 8.7), as well as 29 serum samples from healthy controls (Ctrl, F = 10, M = 19, Age = 66.8 ± 12.6). The patients were recruited as previously described from an ongoing study of lung cancer survival^24^. We selected tumour samples that had at least 70 percent tumour cellularity, with histology of tissue samples confirmed by a pathologist after MRS analyses. With a specific emphasis on studying early stage LuCa, the studied patient population included more low grade Stage I (n = 58, SCC = 27, Adeno = 31, F = 24, M = 34) cases than the more advanced stages (II, III, and IV, n = 35, F = 17, M = 18) combined (Supplementary Table S1 lists patient clinical and demographic information). We further randomly sub-divided Stage I (n = 58) and control (n = 29) cases into Training (SCC = 14, Adeno = 19, Ctrl = 14) and Testing (SCC = 13, Adeno = 12, Ctrl = 15) cohorts and tested when needed. From HRMAS MRS measurements of these samples, we identified 32 spectral regions (width: 0.026 ± 0.010 ppm) that presented measurable spectral intensities in more than 90% of spectra for both tissue and serum samples. We present results using spectral regions, rather than individual metabolites, since each region may include contributions from multiple metabolites, and conversely, a single metabolite can contribute to multiple spectral regions. However, we discuss major possible contributing metabolites when relevant. Principal component analyses (PCA) can be used to reduce data dimensions by identifying PCs that have eigenvalues greater than 1.0 and can be further analysed. PCA performed on these 32 regions for tissue and serum MRS data sets independently produced eight PCs, all with eigenvalues greater than 1.0, for both tissue and serum MRS data sets, respectively. The eight PCs accumulatively represent 79.2% and 77.2% of variance for tissue and serum, respectively. All the statistically significant results presented below were verified by co-variance analyses of age and smoking status (packyear).

Results presented here will include the following four aspects: (1) serum MRS data that differentiate LuCa from healthy controls and among LuCa types and stages; (2) tissue MRS data that differentiate LuCa types and stages; (3) correlations between serum and tissue MRS measurements; and (4) predictions of LuCa overall survival with metabolomics.

### Serum MRS – identifying LuCa from controls and differentiating LuCa types and stages

We defined the serum relative spectral intensity, RelInt(Ser), for spectral region m (m = 1, 2, … 32) and samples = 1, 2, … 93 as:

1 $${{\rm{RelInt}}}_{{\rm{m}},{\rm{s}}}({\rm{Ser}})( \% )=({\mathrm{Exp}}_{{\rm{m}},{\rm{s}}})\times 100/\sum _{i=1}^{32}(Ex{p}_{i,s})$$

where, (Exp_m,s_) represents the experimental value for spectral region m and $\sum _{i=1}^{32}(Ex{p}_{i,s})$ represents the sum of measured values for all 32 spectral regions, for each of the 93 samples.

**Figure 1:**
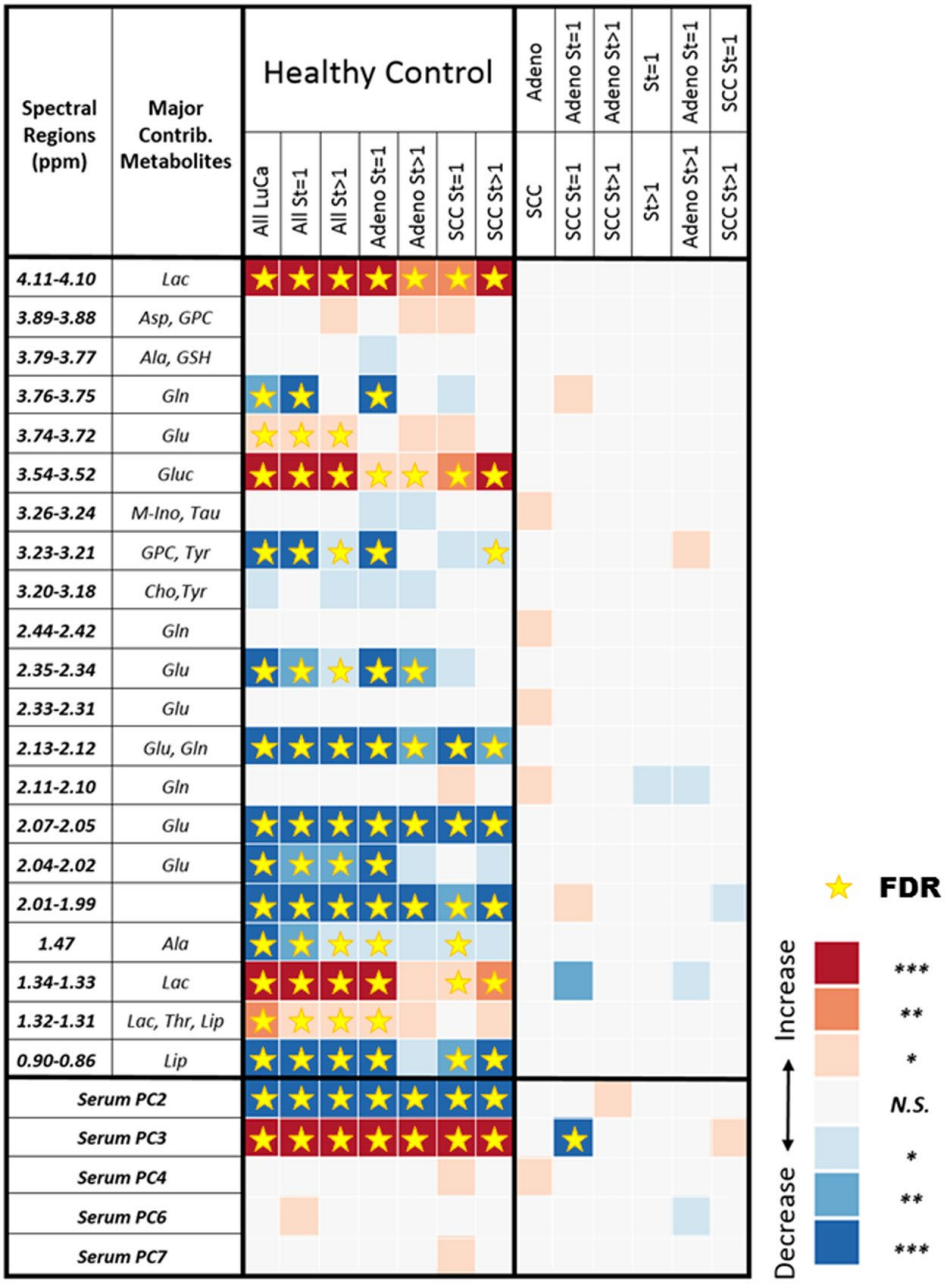
Serum MRS identification of LuCa from controls and differentiation among LuCa types and stages. LuCa groups vs. healthy controls (central column) and various LuCa types and stage groups (right column) were compared with two-tailed Student’s t-test or Mann-Whitney-Wilcoxon test. “Increase” indicates that the values are higher in the lower group compared to the upper group; “Decrease”, indicates the opposite. For instance, the first red square in the table shows that the group of all LuCa samples presented significantly higher serum lactate (Lac, 4.11–4.10 ppm) than the group of healthy controls. See Supplementary Table S1 for sample numbers in each group, and see text for significance notation details. Abbreviations: Ala, alanine; Asp, aspartate; Cho, choline; Glc, glucose; Gln; glutamine; Glu, glutamate; GPC, glycerophosphocholine; GSH, glutathione; Lac, lactate; Lip: lipids; M-Ino, myo-inositol; PC, principal component; St, stage; Tau, taurine; Thr, threonine; Tyr, tyrosine; Val, valine. The notations of statistical significance levels are: “*”p < 0.05; “**”p < 0.005; and “***”, Bonferroni-corrected thresholds of statistical significance of p < 0.0016 or p < 0.0063 for 32 individual regions or 8 PCs, respectively. The star symbols denote statistical significances after FDR calibration.

Results from serum MRS showed significant differences in spectral relative intensities between groups of interest. Figure 1 summarizes the observed statistical significances of relative spectral intensities for 19 among 32 analysed spectral regions and 5 out of 8 PCs measured from serum samples that differentiate healthy controls from different LuCa groups (central column), as well as 8 spectral regions and 4 PCs to differentiate among different groups of LuCa types and stages (right column), according to Student’s t-test (for normal distributions with or without equal variance) or Mann-Whitney-Wilcoxon test (for non-normal distributions). The notations of statistical significance levels in Fig. 1, and for the rest of the report, are as follows: “*”p < 0.05; “**”p < 0.005; and “***”, Bonferroni-corrected thresholds of statistical significance of p < 0.0016 or p < 0.0063 for 32 individual regions or 8 principal components (PC), respectively. The star symbols in this figure, and figures hereafter, denote statistical significance values after calibration with false discovery rate (FDR) analyses.

**Figure 2:**
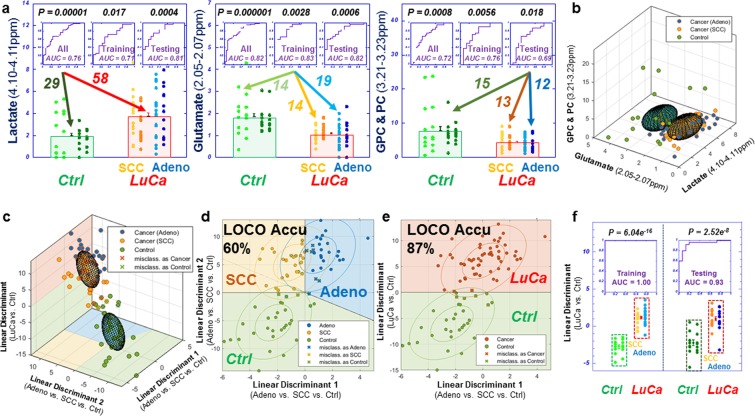
Comparisons between analyses with serum MRS metabolites and analyses with metabolomics. (**a**) Panels of three metabolites that can significantly differentiate LuCa from control groups both for all the tested Stage I, SCC (n = 27) and Adeno (n = 31), and control (n = 29) cases, and for the Training and Testing cohorts, separately. (**b**) The 3D ellipsoids generated from the three panels in (**a**) that cover the volume of 3D Mahalanobis distance ≤1 (one standard deviation from the centroid along each axis) from the class means for LuCa and control groups, according to the covariance matrix of all class-mean-corrected samples. (**c**) Two-class (LuCa vs. control) and three-class (Adeno vs. SCC vs. control) linear discriminations calculated from 19 spectral regions in Fig. 1. Misclassifications are indicated by X’s. (**d**,**e**) 2D projections for three-class and two-class LD results from (**c**). The ellipses represent the 2D Mahalanobis distances 1 and 2 standard deviations from the class means, respectively. Solid lines on projection planes represent active decision boundaries. Classification accuracies shown are after leave-one-out cross-validation. (**f**) Differentiations between LuCa and control cases calculated with LD canonical correlation analysis for the Testing cohort by using analytic parameters obtained from the Training cohort.

Multiple spectral regions showed statistical significance in differentiating LuCa from controls in Fig. 1. In Fig. 2a, Stage I are compared with control cases with three panels presenting examples of three significant regions: lactate (4.10–4.11 ppm), glutamate (2.05–2.07 ppm), and GPC & PC (3.21–3.23 ppm). While these metabolic regions can significantly differentiate Stage I LuCa cases from controls both for the entire tested populations, and for Training and Testing cohorts, respectively, overlap between control and LuCa samples is also obvious, represented by modest receiver operating characteristic (ROC) curves (area-under-curve, AUC = 71~83%), as well as by the closeness of the two 3D ellipsoids presented in Fig. 2b. Invoking the metabolomic concept of multi-dimensional comparisons (in contrast to a single metabolite evaluation), leave-one-case-out (LOCO) cross-validated linear discriminator (LD) analyses involving all 19 spectral regions presented in Fig. 1, in Fig. 2c, Drastically improved differentiations between LuCa and control (vertical panel) presented as well separated 3D ellipsoids (ROC AUC = 98.9%), as well as among all three groups (horizontal panel) were observed. Figure 2d,e further detail metabolomic differentiation among all three groups and between LuCa and controls, respectively. The indication of the existence of metabolomic differentiations between LuCa and controls led us to further test the validity of the observation with the above defined Training and Testing cohorts. Figure 2f demonstrates the significant LuCa and control differentiation results measured with LD canonical correlation analysis for the Testing cohort by using analytic parameters obtained from the Training cohort.

### Tissue MRS – differentiating LuCa types and stages

Unlike serum samples, which are homogenous fluids, tissues are heterogeneous mixtures that are comprised of both diseased and healthy pathological components. Metabolite levels vary in different pathological features, so tissue MRS results must be interpreted in the context of tissue pathologies. The most significant advantage of HRMAS MRS – its ability to preserve tissue architecture for subsequent pathological evaluations – enables us to conduct pathological analyses after MRS measurement to calibrate contributions of tissue pathologies, and their inherent metabolic differences, towards the observed MRS values. For the studied LuCa tissues, four major pathology features were quantified for each specimen after HRMAS MRS: vol% of LuCa (in 58/93 measured samples, with Max: 94.9%, Median: 26.1%), Fibrosis/Inflammation (FI, 89/93, Max: 100%, Median: 74.4%), Necrosis (Nec, 31/93, Max: 100%, Median: 25%), and Cartilage/Normal (CN, 8/93, Max: 89.7%, Median: 47.5%).

We determine the relationship between tissue MRS and pathologies using a least-square regression of an over-determined linear model (LSR-ODLM), which includes 93 linear equations comprised of four pathology features and the experimentally measured value (Exp_m,s_) for all 32 spectral regions m according to the following equation, for samples s = 1, 2, … 93:

2 $$\begin{array}{c}[{{\rm{C}}}_{{\rm{LuCa}},{\rm{m}}}\times {\rm{LuCa}}{ \% }_{{\rm{s}}}]+[{{\rm{C}}}_{{\rm{FI}},{\rm{m}}}\times {\rm{FI}}{ \% }_{{\rm{s}}}]+[{{\rm{C}}}_{{\rm{Nec}},{\rm{m}}}\times {\rm{Nec}}{ \% }_{{\rm{s}}}]\\ \,+[{{\rm{C}}}_{{\rm{CN}},{\rm{m}}}\times {\rm{CN}}{ \% }_{{\rm{s}}}]+{{\rm{a}}}_{{\rm{m}}}={\mathrm{Exp}}_{{\rm{m}},{\rm{s}}},\end{array}$$

where the contribution coefficients (C_x,m_) of pathology feature (x = LuCa, FI, Nec, and CN) percentage towards the experimental value in region m are determined solely from the spectral data without any additional assumptions or weighting for the quantified pathological features; a_m_ is a spectral region-specific constant. The contribution coefficients from each of these four pathological features for the 32 analysed regions can be found in Supplementary Fig. S1.

To evaluate these coefficients, we calculated the estimated spectral intensity (Est_m,s_) for each spectral region, m, and each sample, s, based on the pathological compositions of the sample and the contribution coefficients:

3 $$\begin{array}{c}[{{\rm{C}}}_{{\rm{LuCa}},{\rm{m}}}\times {\rm{LuCa}}{ \% }_{{\rm{s}}}]+[{{\rm{C}}}_{{\rm{FI}},{\rm{m}}}\times {\rm{FI}}{ \% }_{{\rm{s}}}]+[{{\rm{C}}}_{{\rm{Nec}},{\rm{m}}}\times {\rm{Nec}}{ \% }_{{\rm{s}}}]\\ \,+[{{\rm{C}}}_{{\rm{CN}},{\rm{m}}}\times {\rm{CN}}{ \% }_{{\rm{s}}}]+{{\rm{a}}}_{{\rm{m}}}={{\rm{Est}}}_{{\rm{m}},{\rm{s}}}\end{array}$$

After calibration for pathology contributions, the difference between Exp_m_ and Est_m_, Exp_m_ − Est_m_, can be considered to be independent from tissue pathological compositions. This is supported by the comparisons of the linear regression analyses conducted between tissue pathological compositions (vol%) and Exp_m_, as well as (Exp_m_ − Est_m_) values. For instance, the results of linear regression analyses evaluated between LuCa vol% and Exp_m_ values for tissue samples presented statistically significant (p < 0.050) correlations for 22 out of the 32 spectral regions, with p values ranging from <0.001 to 0.045 (mean = 0.010 ± 0.003). However, when linear regression analyses were evaluated between LuCa vol% and (Exp_m_ − Est_m_) values, no significant linear correlation was seen. The p values for the same 22 regions were determined to be between 0.110 and 1.00 (mean = 0.710 ± 0.074).

Therefore, the values of Exp_m_ − Est_m_, or the values of Exp_m_/Est_m_ (to avoid negative values), after calibration of the tissue pathological contributions, could be attributed to tissue MRS results that are largely reflecting patient disease status rather than pathology features. The total calibrated spectral intensity from 32 regions for sample s is:

4 $${\rm{Total}}\,{{\rm{Int}}}_{{\rm{s}}}({\rm{Tis}})=\sum _{i=1}^{32}(Ex{p}_{i,s}/Es{t}_{i,s}).$$

Then, the calibrated tissue relative spectral intensity, RelInt_m,s_(Tis), for spectral region m and samples s is:

5 $${{\rm{RelInt}}}_{{\rm{m}},{\rm{s}}}({\rm{Tis}})( \% )=({\mathrm{Exp}}_{{\rm{m}},{\rm{s}}}{/\mathrm{Est}}_{{\rm{m}},{\rm{s}}})\times 100/{{\rm{TotalInt}}}_{{\rm{s}}}({\rm{Tis}}).$$

**Figure 3:**
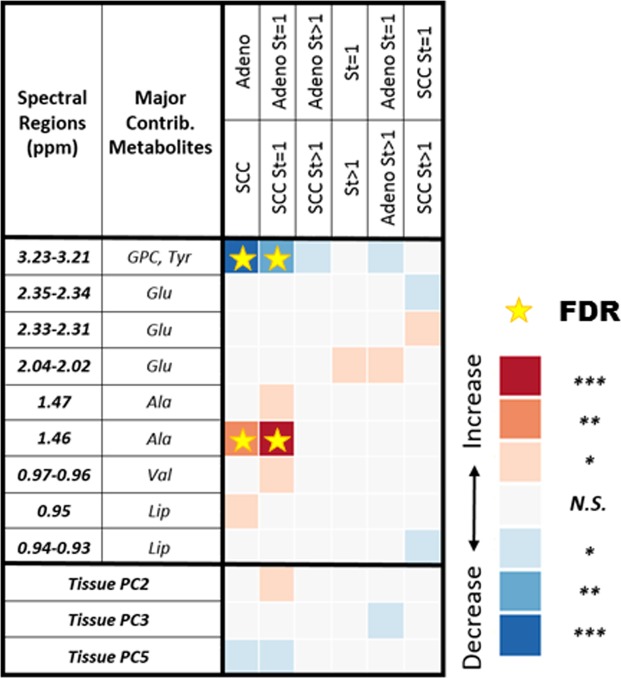
Tissue MRS differentiations among LuCa types and stages. After pathology calibrations for the tissue MRS spectral data, various LuCa types and stage groups were compared with two-sided Student’s t-test (for normal spectral region distributions) or Mann-Whitney-Wilcoxon test (for non-normal spectral region distributions). “Increase” indicates that the values are higher in the lower group compared to the upper group; “Decrease”, indicates the opposite. For instance, the first blue square in the table shows that the group of SCC samples presented significantly lower spectral intensity in the glycerophosphocholine and tyrosine region. See text for significance notation details, and see Supplementary Table S1 for sample numbers in each group. Abbreviations: Ala, alanine; Glu, glutamate; GPC, glycerophosphocholine; Lip: lipids; PC, principal component; St, stage; Tyr, tyrosine; Val, valine.

**Figure 4:**
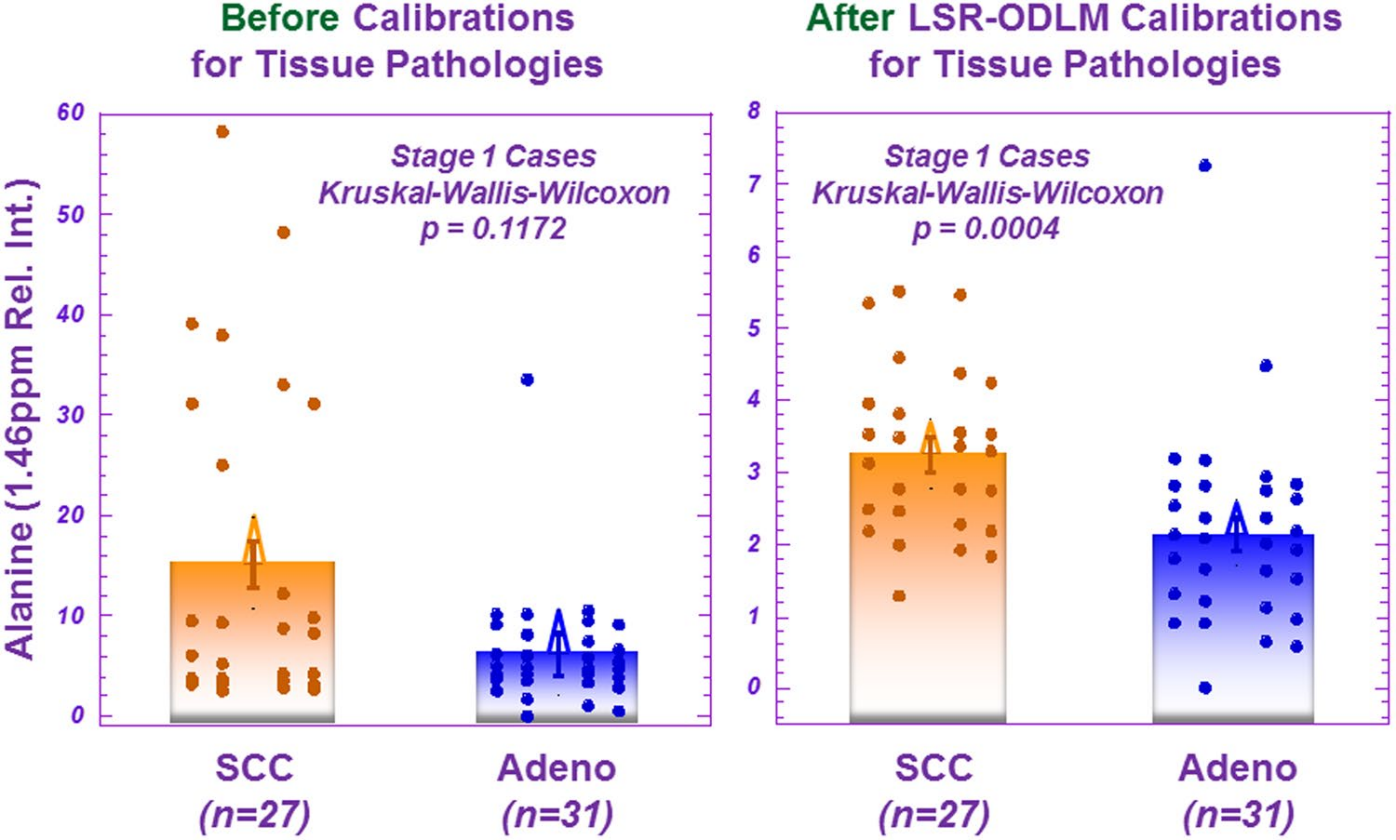
The effects of pathology calibrations on the measured tissue MRS data. Before a least squares regression of an over-determined linear model (LSR-ODLM) pathology calibration for tissue MRS spectral data, the spectral region containing alanine could not distinguish the two groups in a Mann-Whitney-Wilcoxon comparison between Stage 1 SCC (n = 27) 15.234 ± 2.287 and Adeno (n = 31) 6.273 ± 2.134 (relative intensity) cases. After calibrating the MRS spectral data for different percentages of pathology as described by Eqs 2–5, the alanine spectral region, among others, can significantly differentiate between the groups, 3.272 ± 0.241 (SCC) and 2.166 ± 0.225 (relative intensity) (p = 0.0004).

Using the same conventions established in Fig. 1 for serum results, Fig. 3 presents differentiation of LuCa groups according to tissue pathological feature-calibrated MRS results as defined in Eq. 5. Here, 9 of 32 spectral regions present significant differentiation among various LuCa groups. The effects of pathology calibrations on tissue metabolites can be appreciated by an example of alanine (Ala) differentiating Stage I SCC from Adeno groups, as shown in Fig. 4, where significant differentiation was only observed after the applied pathological feature calibration.

### Correlating serum and tissue metabolomic profiles for LuCa differentiation

Studying tissue-serum pairs from the same patient enabled us to use the tissue data set as a training cohort to investigate correlations between serum and tissue MRS data.

**Figure 5:**
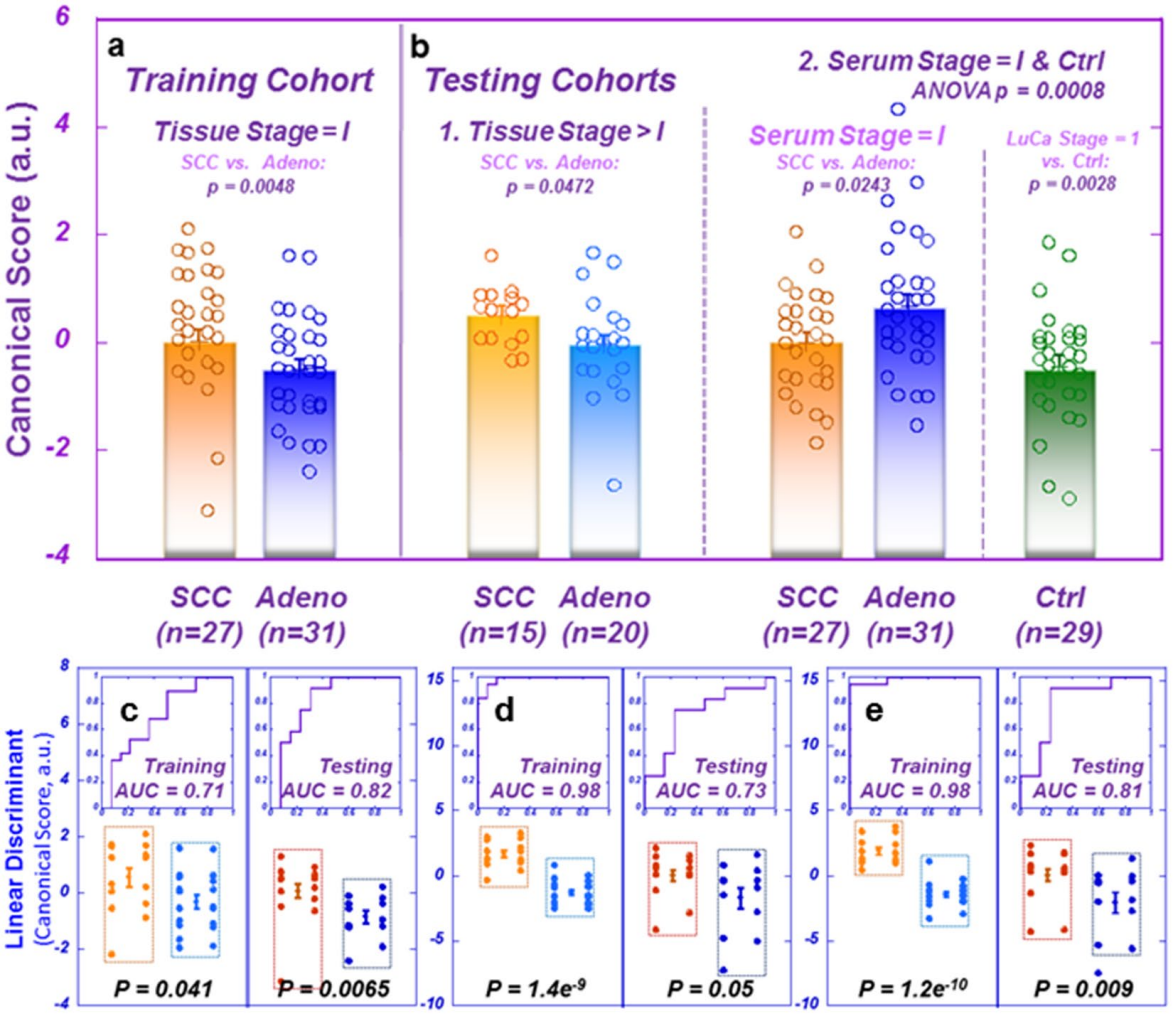
Differentiation between LuCa types and stages with tissue-derived serum metabolomic profiles. (**a**) Training cohort: ANOVA comparison between canonical scores (arbitrary units, a.u.) of Stage I LuCa cases SCC (n = 27; Mean = 0.033 ± 0.208) vs. Adeno: (n = 31; Mean = −0.503 ± 0.194). (**b**) Testing cohorts: (1) ANOVA comparison between canonical scores (a.u.) of tissues from LuCa cases of Stages II, III, and IV SCC (n = 15; Mean = 0.495 ± 0.211) vs. Adeno (n = 20; Mean = −0.038 ± 0.182) and (2) ANOVA comparison between canonical scores (a.u.) of serum PCs calculated with tissue PCA loadings for Stage I cases SCC (n = 27; Mean = −0.010 ± 0.213) vs. Adeno (n = 31; Mean = 0.682 ± 0.199) vs. controls (n = 29; Mean = −0.436 ± 0.206). Given our hypothesis from the tissue training cohort result that mean canonical score values were higher for SCC than for Adeno, a one-tailed test was used for the tissue testing cohort only. (**c**,**d**) Linear discriminant canonical correlation analyses with the 19 spectral regions (cf. Fig. 1), for tissue and serum MRS data of the Training cohort, respectively. The capabilities of the resulting canonical scores in differentiating SCC from Adeno groups were presented with the results obtained from the Testing cohort. (**e**) Linear discriminant canonical correlation analyses including both tissue and serum results (from **c**,**d**), increased statistical significance for SCC and Adeno differentiations.

As previously described, the PCA conducted on tissue spectra data set with all 32 spectral regions from 93 samples produced eight PCs with eigenvalues greater than 1.0 that represented 79.2% of the total variance of the data set. To emphasize characterization of early stage LuCa, we performed a canonical correlation analysis (CCA) using these eight PCs from 58 Stage I LuCa cases (SCC = 27 and Adeno = 31) as a training cohort to discover a discriminator that can differentiate Adeno from SCC for Stage I LuCa (Fig. 5a). We then applied the obtained CCA loadings to two testing cohorts: first, the tissue data set from LuCa cases of Stage > I (combining Stages II, III, and IV) and second, PCs for Stage I and healthy serum samples, calculated with tissue PCA loadings, as shown in Fig. 5b. Tissue cases with Stages > I of the first testing cohort confirmed the differentiation between SCC and Adeno seen with the training cohort. Serum spectral data of the second testing cohort presented discriminating results between Stage I cases and healthy controls, as well as between Stage I SCC and Adeno cases. However, the score values were higher for SCC in tissues, but significantly lower in sera. This reversal trend seen in differentiations between SCC and Adeno in tissue and serum data sets is caused by an inverse relationship between the SCC/Adeno ratios of the means of relative intensities presented for tissue and serum (see Supplementary Fig. S3) and the combined PCA and CCA loadings from tissue (Supplementary Table S2).

The successes demonstrated in Fig. 5a,b, further guided us to test with randomly determined Training and Testing cohorts, including Stage I LuCa and control cases, previously presented with Fig. 2a,f. In Fig. 5c,d, linear discriminant canonical correlation analyses were conducted with the 19 spectral regions (Fig. 1), for tissue and serum MRS data of the Training cohort, respectively. The capabilities of the resulting canonical scores in differentiating SCC from Adeno groups were tested with the Testing cohort presenting indications of differentiations, but the serum result (p = 0.0502) was just above the level of significance. However, the study design of paired tissue and serum samples obtained from the same patients permitted us to conduct a further canonical analysis including both tissue and serum canonical scores for the Training cohort, and we tested the resulting canonical score on the Testing cohort with improved statistical significance (p = 0.009) when compared with the score obtained from serum data alone.

### Predictions of LuCa overall survival with MRS metabolomics

Clinical records (1997–2012) for the studied LuCa patients indicate the average survival time after surgery to be 41.3 ± 4.6 months (n_Adeno_ = 27, Mean: 43.4 ± 6.4 months, and n_SCC_ = 27, Mean: 39.2 ± 6.7 months). Using 41.3 mo as a threshold to define short vs. prolonged surviving, we observed a number of tissue and serum spectral regions that can differentiate between the two groups. Most importantly, some of these spectral regions from both tissue and serum can further provide statistically significant Kaplan-Meier estimates of 10-year overall patient survival for the entire population, as well as for certain subgroups (see Fig. S4).

**Figure 6:**
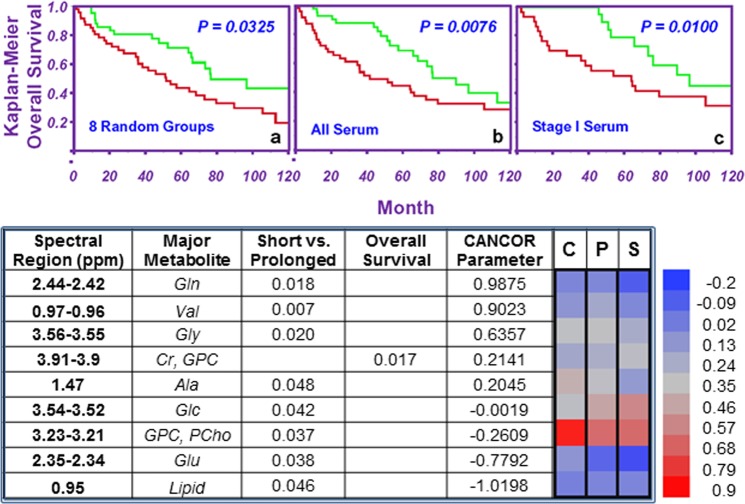
Evaluations of overall survival with serum MRS metabolomics. (**a**) Results of leave-one-group-out validation show the ability of canonical scores to differentiate short living (red, n = 29) from prolonged living (green, n = 25) groups for all 93 cases in 8 cohorts (7 groups = training; left-out group = testing). (**b**,**c**) All 93 cases were combined. Nine spectral regions were identified (**d**) which differentiated survival groups. CCA scores for these regions significantly differentiated 10-year survival for both (**b**) the entire LuCa population and (**c**) Stage I cases alone (red, n = 14; green, n = 18). (**d**) As mentioned, all 93 cases were combined, and the serum spectral regions were evaluated by canonical analysis. Nine spectral regions could differentiate between controls (C), prolonged survival (P), and short survival (S) groups, and one region could identify overall survival. The heatmap presents log spectral intensities to illustrate that the prolonged survival group more closely resembles the short survival group or controls, but that the short survival group and controls are noticeably different.

To systematically evaluate the prognostic potential of serum metabolomics, we randomly divided 93 cases into eight groups (maximum number of cases per group: 14; minimum: 9). We combined seven groups to form the training cohort, and used the one group left out as the corresponding testing cohort. We iterated the leave-one-group-out process eight times to cover all eight groups (i.e. the eight corresponding testing cohorts). For each training cohort, we identified regions among the 32 spectral regions that can differentiate short vs. prolonged surviving groups with statistical significance (p < 0.05). The numbers of spectral regions thus identified ranged from one to seven (average: 3.75 ± 0.88) for the eight training cohorts. For each training cohort, a canonical correlation analysis including the identified spectral regions was first conducted to discover CCA loadings to discriminate short from prolonged surviving groups within the cohort. The loadings obtained from the training cohort were applied to the cases in the corresponding testing cohort to obtain the CCA scores for cases in the testing cohort. Upon the completion of all eight iterations, the CCA scores for all cases obtained when they were considered as testing cohort cases were combined into a single ensemble. The median value for the ensemble was determined and used as the threshold to evaluate the 10-year overall survival based on the Kaplan-Meier curves, which displayed statistical significance between short (red) and prolonged living groups (green) (p = 0.0325), as shown in Fig. 6a. The effects of tissue pathology calibration can also be seen when the measured tissue metabolic intensities are used to predict patient overall survival. For instance, pathology-calibrated spectral intensities of 3.91–3.90 ppm region are sensitive to predicting patient overall survival for the entire tested population, for SCC cases, and particularly for Stage 1 cases of SCC. However, this significant separation for a single disease stage, which cannot be differentiated by currently known clinical parameters, would be invisible without the pathology calibrations (Fig. S4).

While the leave-one-group-out analyses indicated the potential existence of metabolomic discriminators between short and prolonged LuCa patient survival, the method cannot provide a single set of parameters able to evaluate the status of a future case. Nevertheless, this proof of the potential existence of the survival-related intrinsic LuCa metabolomics encouraged us to further analyse all cases in a single data set. With all 93 cases, we identified nine serum spectral regions (Fig. 6d) that show significant differentiation between short and prolonged survival groups. By including these nine spectral regions in a canonical analysis to discriminate (p < 0.0001) short (<41.3 months, n_SCC_ = 15, n_Adeno_ = 14, CCA score = −0.652 ± 0.186; n_SCC,St=I_ = 9, n_Adeno,St=I_ = 5, CCA score = −0.837 ± 0.254) from prolonged (>41.3 months, n_SCC_ = 12, n_Adeno_ = 13, CCA score = 0.756 ± 0.200; n_SCC,St=I_ = 8, n_Adeno,St=I_ = 10, CCA score = 0.766 ± 0.224) survival (Supplementary Table S1), we were able to predict 10-year Kaplan-Meier overall survival estimates for both the entire LuCa population (SCC = 42, Adeno = 51) (Fig. 6b) and the Stage I cases alone (SCC = 27, Adeno = 31) (Fig. 6c) by using their respective median of the canonical scores as the discriminators. Prolonged survival is associated with relative overexpression of Gln, Val, and Gly, and relative suppression of Glu and lipids in serum. This last conclusion obtained from sera of stage I LuCa patients is of critical importance for its potential utility in clinic. At present, the criteria used in the LuCa clinicians for patient assessments are mostly based on clinical experiences accumulated from symptomatic and late-stage patients that cannot be applied to the assessment of asymptomatic patients with early stage disease which is now detected through advanced radiological tests. Thus, new prediction parameters for survival of Stage I LuCa will assist the advancement of the LuCa clinic. The heatmap in Fig. 6d illustrates that metabolite intensities for the prolonged living group (P) are either closer to the control group (C) or the short living group (S), whereas the intensities for C and S groups are noticeably different.

## Discussion

The aim of the current study is to evaluate potential human blood serum LuCa metabolomic markers that may be used to screen high-risk individuals for advanced imaging for detection of LuCa at early and asymptomatic stages. However, while identification of cancer blood screening biomarkers is extremely attractive due to the less invasive nature of specimen collection, research in this area is often challenged by low specificity, since blood circulates throughout the entire body. To associate serum metabolites with LuCa, we designed the study to include paired LuCa tissue and serum samples from the same patients. With such an experimental set-up, metabolites quantified in the serum samples could be investigated in conjunction with those measured from tissue samples.

Each analysed sample, either a tissue specimen of ~10 mg or a drop of serum of ~10 µl, produces a single MRS spectrum. However, a tissue sample, even as small as on a mg scale, represents a mixture of various pathological components, such as cancer, inflammation, fibrosis, and necrosis. Metabolite values are affected by not only the stage of disease from which the tissue is acquired but also the amount of cancerous and other cells present in the tissue. Thus, understanding metabolite concentrations in tissue requires consideration of pathology variations, and HRMAS MRS technology allowed us to quantify these amounts. We then calibrated MRS-measured metabolite values according to varying amounts of pathological components in each sample, rather than merely make qualitative comparisons between pathology percentages and metabolites^25–28^.

This calibration adjustment generated stronger results for tissue and serum analyses, but the interpretation of the observed reversed relationship when comparing SCC with Adeno for tissue and serum data sets (Fig. 5) requires caution. The apparent inverted slopes shown in Supplementary Fig. S3 were presented to explain the reversed relationship seen in Fig. 5a, but are not to be interpreted as the presentation of reversed metabolite concentrations between SCC and Adeno cases when comparing their metabolite concentrations in tissues with those in sera. To compare the selected 32 spectral regions for tissue and serum at a similar intensity level, we elected to analyse relative spectral intensities. With tissue, further calibrations of the relative spectral intensities according to tissue pathological compositions were implemented. Therefore, the above-mentioned apparent inverted slopes represent only the relationship seen with these calibrated relative spectral intensities, and cannot be simply extended to indicate metabolic concentrations. Additional analyses that will allow for quantification of metabolites from different sources will be necessary to understand these observations in detail. Furthermore, since blood is the main nutrient source for all physiological and pathological processes, it cannot simply be viewed as the “dumping ground” of cancer metabolisms active in tissue lesions. Therefore, the metabolomic profiles presented by blood serum cannot be expected to mimic those measured from cancerous tissues. Nevertheless, analyses of the similarities and differences of cancer metabolomic profiles measured from paired tissue and serum samples will improve understanding of cancer metabolism both for patient prognostication and design of treatment strategies.

MRS measurements of tissue and serum samples present snapshots of cellular metabolites. In the case of blood, metabolite levels measured in a LuCa patient may reflect altered output or uptake by cancer cells. For instance, when comparing serum profiles between prolonged and short survival groups, the prolonged survival cases favoured elevated expressions of glutamine, valine, and glycine (positive CCA loadings), and suppressed expressions in glutamate and lipid droplets (negative CCA loadings). The alterations of glutamine and glutamate may be interpreted through their metabolic mechanism. Glutamine is an essential metabolite to support anabolic metabolism in tumour cells^29^, and high consumption of glutamine has been reported for cancer cells^30^. Cancer cells use the enzyme glutaminase to convert glutamine to glutamate, and to form precursors for the processes of anaplerosis, glutathione synthesis, and fatty acid production, which allow for tumorigenesis^31^. Glutamine itself is also important energy source for cancer cells when glucose availability is limited^32^. These biological realities of cancer support the finding of higher glutamine in prolonged cases. Furthermore, blood maintains high levels of glutamine as a ready source of carbon and nitrogen to support biosynthesis, energetics, and cellular homeostasis, and cancer cells may hijack this supply for tumour growth^33^, which may particularly be true in the cases of fast-growing LuCa of the short survival cases. On the other hand, in prolonged survival cases (which closely match the healthy controls for glutamine levels in (Fig. 6d) the consumption of glutamine, for conversion to glutamate, is less than consumption by the short survival cases. Less glutamine consumption results in less production of lipid droplets and glutamate for these longer-surviving cancer cases. Similarly, for the essential amino acid valine, studies have shown that non-small cell LuCa tumours displayed a significant increase in valine uptake^34^. Thus, the elevated blood valine levels seen in the prolonged survival cases may present less uptake of valine when compared with short survival cases. The same reasoning can be extended to the observed elevated serum glycine levels when comparing prolonged with short survival cases, where glycine provides the carbon units to fuel the one-carbon metabolism for the synthesis of proteins, lipids, nucleic acids, etc.^30^. The observation of the association between higher levels of glycine and poorer prognoses reported for human breast cancer agree well with our measurements described here^35^.

In this exploratory study, our current results are limited by the scale of the study. First, we only analysed tissue and serum samples from a LuCa tumour bank enrolling cancer-positive patients who presented with symptoms or whose cancer was found incidentally. The biomarkers thus obtained may only apply to the studied patient populations, and may not be extrapolated to other patient populations, such as asymptomatic patients. Second, this study only investigated two major types of non-small-cell LuCa, so again the conclusions are not applicable to other types of LuCa without further validation on enlarged patient populations.

Nevertheless, our proof-of-concept MRS exploratory study of paired human LuCa tissue and serum samples demonstrates the potential of a physical chemistry approach for the discovery of human serum LuCa metabolomic markers. While the reported LuCa markers have been tested under a training and testing cohort design, the limitations of small case numbers and the need for analysing more diverse patient populations argue for more comprehensive studies to be conducted. Success in these investigations can propel biomarkers towards clinical trials and towards the ultimate goal – to indicate cancer and screen patients to advanced radiological imaging when warranted.

## Materials and Methods

### Study design

#### Experimental design

This study was approved by the Partners Human Research IRB (Protocol 2009P000982), and all research was performed in accordance with relevant guidelines and regulations. Serum and tissue samples were obtained from the Harvard/MGH Lung Cancer Susceptibility Study Repository. Informed consent was obtained from LuCa patients and healthy controls prior to banking samples and after the nature and possible consequences of the study were explained. The objective of this retrospective, paired tissue-serum investigation was to discover biomarkers in LuCa tissue of early stage LuCa which can also be measured in serum. Based on our initial, preliminary evaluation of lung cancer biomarkers published previously^36^, we designed this exploratory study to analyse ~100 samples. After evaluation of 101 samples, it was determined that the spectral resolution for 8 samples was not sufficient for further analysis, and only 93 were included in the current study.

#### Study population

Patient information: Detailed information on the studied patient population can be found in Supplementary Table S1. Researchers were blinded to the status of the samples during all measurement and experimental steps.

#### Intact tissue MRS

Samples were stored at −80 °C until analysis. High resolution magic angle spinning magnetic resonance spectroscopy (HRMAS MRS) measurements were performed using our previously developed method on a Bruker Avance (Billerica, MA) 600 MHz spectrometer. Measurements were conducted at 4 °C with a spin-rate of 3600 ± 2 Hz and a Carr-Purcell-Meiboom-Gill (CPMG) sequence with and without continuous-wave water suppression. Ten µL of serum or 10 mg of tissue were placed in a 4 mm Kel-F zirconia rotor with 10 uL of D_2_O added for field locking. HRMAS MRS spectra were processed using a laboratory developed MATLAB-based program, and peak intensities from 4.5–0.5 ppm were curve fit. Relative intensity values were obtained by normalizing peak intensities by the total spectral intensity between 4.5–0.5 ppm. The resulting values that were less than 1% of the median of all curve fit values were considered as noise and eliminated. Spectral regions were defined by regions where 90% or more of samples had a detectable value, resulting in 32 regions.

#### Quantitative histopathology

Following MRS measurement, tissues were formalin-fixed and paraffin-embedded. Serial sectioning was performed by cutting 5 µm-thick slices at 100 µm intervals throughout the tissue, resulting in 10–15 slides per piece. After hematoxylin and eosin (H&E) staining, a pathologist with >25 years experience read the slides to the closest 10% for percentages of the following pathological features: cancer, inflammation/fibrosis, necrosis, and cartilage/normal.

### Statistical analysis

Statistical analyses were performed using JMP Pro 13 and MATLAB 2017a. Univariate statistical tests included Student’s t-test (for spectral regions with normal distribution according to Shapiro-Wilk W test) or Mann-Whitney-Wilcoxon test (MWW, for spectral regions with non-normal distributions) for binary comparisons; analysis of variance (ANOVA, for normal distributions) or Kruskal-Wallis-Wilcoxon (KWW, for non-normal distributions) for ≥ ternary comparisons. Multivariate analyses included principal component analysis, linear discriminator analysis, and canonical correlation analysis. Associations between canonical correlation scores and survival were assessed using Kaplan-Meier survival curves and log-rank tests. Additionally, MRS spectral measurements from tissue were calibrated to account for the contributions from varying amounts of pathological components in each sample, using a least squares regression-over-determined linear regression model. In addition to reporting comparisons with an alpha level = 0.05, false discovery rate (FDR) analysis and Bonferroni corrections to account for multiple testing of 32 spectral regions and 8 principal components were invoked. Except where noted and explained, two-sided testing was used. All the statistically significant results presented were verified by co-variance analyses of age and smoking status (packyear).

## Supplementary information

Magnetic Resonance Spectroscopy-based Metabolomic Biomarkers for Typing, Staging, and Survival Estimation of Early-Stage Human Lung Cancer

## Data Availability

Data reported in this paper are available at our repository through the Martinos Center (http://www.nmr.mgh.harvard.edu/~cheng/MRSbenign/).
