# Supplementary material for: Magnetic Resonance Spectroscopy-based Metabolomic Biomarkers for Typing, Staging, and Survival Estimation of Early-Stage Human Lung Cancer
Source: Sci Rep. 2019 Jul 16;9:10319. doi: 10.1038/s41598-019-46643-5 (PMC6635503; doi:10.1038/s41598-019-46643-5)
Supplement: Supplementary file 1 — Magnetic Resonance Spectroscopy-based Metabolomic Biomarkers for Typing, Staging, and Survival Estimation of Early-Stage Human Lung Cancer [file 41598_2019_46643_MOESM1_ESM.pdf]

Magnetic Resonance Spectroscopy-based Metabolomic Biomarkers for Typing, Staging, and Survival Estimation of Early-Stage Human Lung Cancer

Yannick Berker[1,2,3,†], Lindsey A. Vandergrift[1,2,†], Isabel Wagner[1,2,4], Li Su[5], Johannes Kurth[1,2,6], Andreas Schuler[1,2], Sarah S. Dinges[1,2,6], Piet Habbel[6], Johannes Nowak[7], Eugene Mark[2], Martin J. Aryee[2,8], David C. Christiani[5,*], Leo L. Cheng[1,2,*]

[1]Department of Radiology, Massachusetts General Hospital, Harvard Medical School, Boston, Massachusetts, 02114 USA.

[2]Department of Pathology, Massachusetts General Hospital, Harvard Medical School, Boston, Massachusetts, 02114 USA.

[3]Division of X-Ray Imaging and Computed Tomography, German Cancer Research Center (DKFZ), 69120 Heidelberg, Germany.

[4]Department of Urology, CCM, Charité – Universitätsmedizin Berlin, Chariteplatz 1, 10117 Berlin, Germany.

[5]Department of Environmental Health, Harvard T.H. Chan School of Public Health and Department of Medicine, Massachusetts General Hospital/Harvard Medical School, Boston, Massachusetts, 02115 USA.

[6]Department of Haematology and Oncology, CCM, Charité – Universitätsmedizin Berlin, Chariteplatz 1, 10117 Berlin, Germany.

[7]Department of Diagnostic and Interventional Radiology, University Hospital of Würzburg, 97080 Würzburg, Germany.

[8]Department of Biostatistics, Harvard T.H. Chan School of Public Health, 677 Huntington Ave. Boston, MA 02115, USA.

[†]Contributed equally.

[*]**Corresponding Authors**:

Leo L. Cheng, PhD

149 13th Street, CNY-6
Charlestown, MA 02129
Ph. 617-724-6593
Fax. 617-726-5684
cheng@nmr.mgh.harvard.edu

David C. Christiani, MD, PhD

665 Huntington Avenue, Building I, Room 1401
Boston, MA 02115
Ph. 617-432-3323
Fax. 617-726-2932
dchris@hsph.harvard.edu

Supplementary Materials

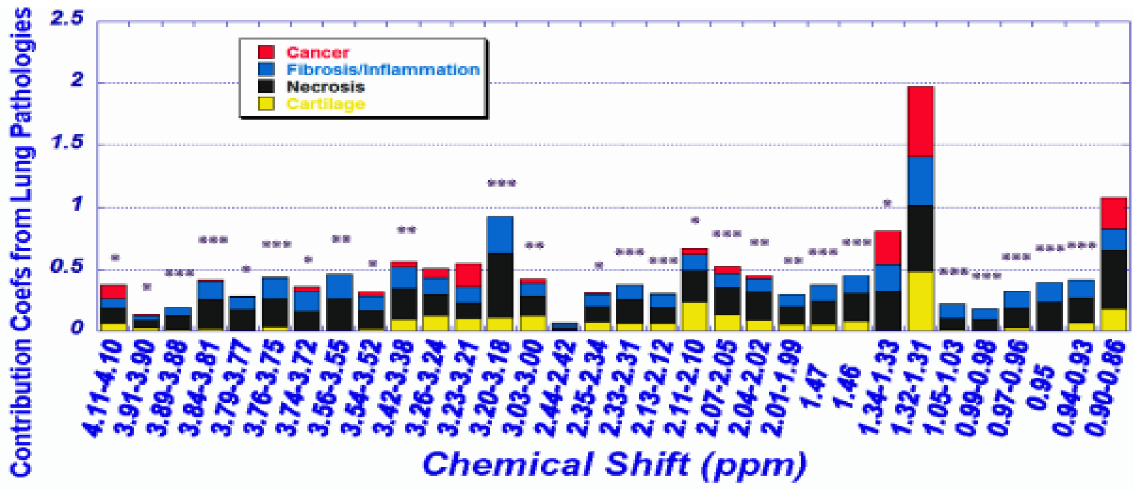

Figure S1. The contribution coefficients of four quantified pathological features for the 32 analysed spectral regions. Estimated spectral intensity ($Est_{m,s}$) was calculated for each spectral region as shown in Eq. 3. The significance levels indicated in the figure represent the levels of agreements between values of Est_m and Exp_m of each spectral region for the 93 tissue samples according to linear regression analyses.

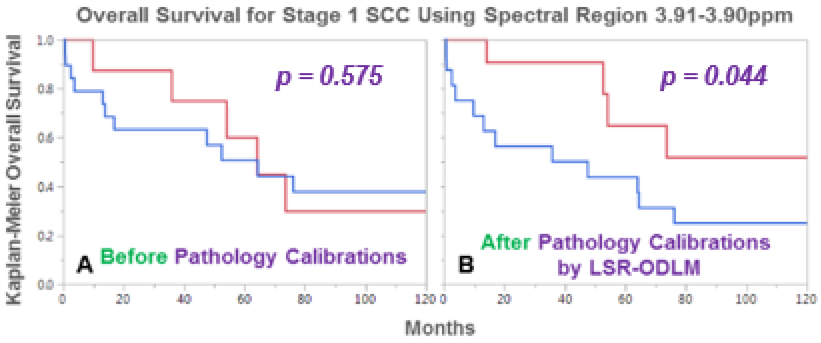

Figure S2. Tissue-calibration effects on MRS estimations of the overall survival for SCC stage 1 patients. Red indicates prolonged living group (n=25); blue indicates short living group (n=29).

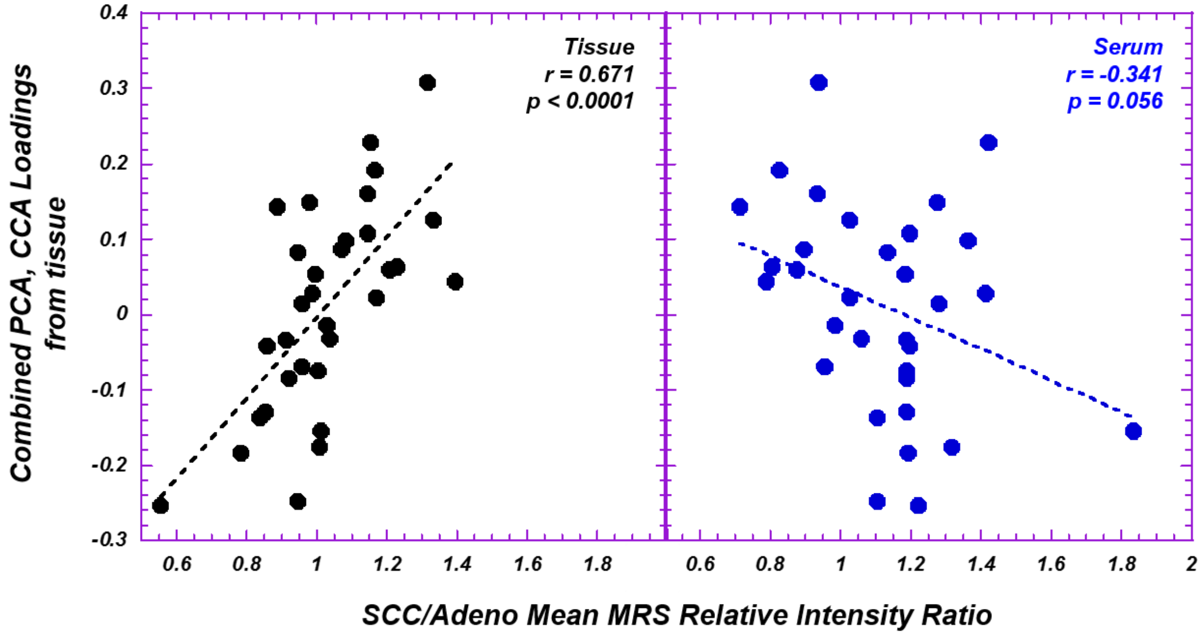

Figure S3. Inverse relationships exist between the tissue PCA and CCA loadings and SCC/Adeno intensity ratios for each metabolic region (represented by a dot) for tissue and serum, respectively. For tissue (top), the positive correlation between the loadings and SCC/Adeno mean relative intensity ratios is expected, given that the canonical correlation analysis using tissue MRS results generated discriminators to separate SCC from Adeno. Specifically, CCA positively weights the ratios which are increased (higher MRS-measured values) in SCC and negatively weights the variables which are decreased (lower MRS-measured values). For serum (bottom), the opposite relationship is observed between the loadings and SCC/Adeno mean relative intensity ratios. These inverse relationships explain the opposite relationship seen in Figure 3B, where SCC has a higher mean canonical score in tissue and lower mean canonical score in serum.

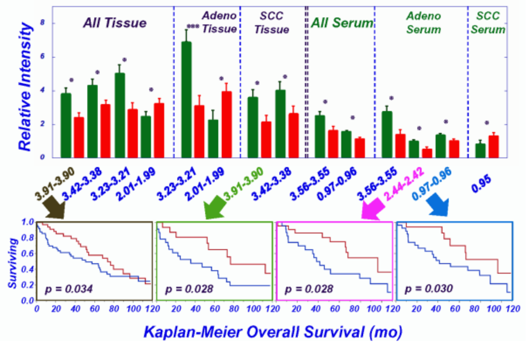

Figure S4. Estimations of LuCa overall survival with tissue and serum MRS data. Mann-Whitney-Wilcoxon comparison between prolonged (red, n=25) and short (blue, n=29) living groups for both tissue and serum spectral data showed multiple spectral regions could differentiate between the groups. The insets show Kaplan-Meier survival curves for four of these spectral regions that can significantly differentiate between the living groups.

Table S1. Data of Patient Population.

		Male		Female	
LuCa Stage		*I*	*>I* (II, III, IV)	*I*	*>I* (II, III, IV)
Adenocarcinomas					
Cases Numbers (#)		***17***	***8***	***14***	***12***
Age (Yr)		*63.55±2.03*	*61.80±3.47*	*63.91±2.29*	*64.90±2.71*
Alive (#, Mo)		***8***, *48.53±14.44*	***3***, *17.73±9.06*	***8***, *82.72±11.65*	***5***, *81.99±22.26*
Death(#, Mo)		***9***, *57.58±12.19*	***5***, *17.89±7.28*	***6***, *58.34±14.43*	***7***, *30.77±8.95*
Smoking Status	*Never*	***1***			
	Former (#, pkyr)	***8***, *38.19±10.98*	***4***, *69.07±20.24*	***7***, *46.64±10.09*	***5***, *51.09±4.23*
	Current (#, pkyr)	***8***, *57.18±12.13*	***4***, *51.26±15.96*	***7***, *43.89±3.50*	***7***, *75.13±14.20*
Squamous Cell Carcinomas					
Cases Numbers (#)		***17***	***10***	***10***	***5***
Age (Yr)		*68.32±2.23*	*67.55±2.95*	*67.55±2.50*	*66.41±2.15*
Alive (#, Mo)		***5***, *93.67±16.59*	***1***, *32.03*	***5***, *88.73±22.20*	***3***, *82.42±7.14*
Death (#, Mo)		***12***, *39.11±11.32*	***8***, *47.57±13.46*	***5***, *36.09±10.41*	***2***, *14.37±10.57*
Smoking Status	*Never*				
	Former (#, pkyr)	***9***, *57.36±12.28*	***6***, *55.27±10.73*	***5***, *81.30±13.89*	***3***, *59.50±12.56*
	Current (#, pkyr)	***8***, *81.83±16.17*	***4***, *82.00±14.05*	***5***, *87.28±25.78*	***2***, *61.14±34.10*
Healthy Controls					
Cases Numbers (#)		***19***		***10***	
Age (Yr)		*68.81±2.97*		*63.06±3.68*	
Smoking Status	*Never (#)*	***3***			
	Former (#, pkyr)	***7***, *47.88±11.46*		***5***, *25.06±10.41*	
	Current (#, pkyr)	***9***, *66.48±16.96*		***5***, *39.37±15.64*	

Table S2. Overall coefficients for LuCa type differentiation.

ppm	*Relevant major contributing metabolites*	*Combined PCA, CCA loadings from tissue*
1.46	*Ala*	0.309
2.33-2.31	*Glu*	0.229
4.11-4.1	*Lac*	0.192
1.47	*Ala*	0.161
3.76-3.75	*Gln*	0.149
1.34-1.33	*Lac*	0.144
0.95	*Lipid*	0.126
2.13-2.12	*Glu, Gln*	0.109
2.44-2.42	*Gln*	0.099
1.32-1.31	*Lac, Thr, Lipid*	0.087
3.84-3.81	*Glc*	0.084
0.99-0.98	*Lipid*	0.064
0.97-0.96	*Val*	0.060
3.89-3.88	*Asp, GPC*	0.054
1.05-1.03	*Val*	0.043
2.01-1.99	*(unknown)*	0.030
3.74-3.72	*Glu*	0.022
2.11-2.1	*Gln*	0.014
0.94-0.93	*Lipid*	-0.014
3.56-3.55	*Gly*	-0.031
2.07-2.05	*Glu*	-0.034
2.04-2.02	*Glu*	-0.041
0.9-0.86	*Lipid*	-0.069
3.03-3	*Cr, PCr*	-0.074

3.79-3.77	*Ala, GSH*	-0.085
3.26-3.24	*m-Ino, Tau*	-0.128
3.42-3.38	*Glc, Tau*	-0.138
3.2-3.18	*Cho, Tyr*	-0.153
2.35-2.34	*Glu*	-0.176
3.91-3.9	*Cr, GPC*	-0.183
3.54-3.52	*Glc*	-0.247
3.23-3.21	*GPC, PCho*	-0.253
